# Podocin is translocated to cytoplasm in puromycin aminonucleoside nephrosis rats and in poor-prognosis patients with IgA nephropathy

**DOI:** 10.1007/s00441-014-2100-9

**Published:** 2015-02-13

**Authors:** Hiromitsu Fukuda, Teruo Hidaka, Miyuki Takagi-Akiba, Koichiro Ichimura, Juan Alejandro Oliva Trejo, Yu Sasaki, Juan Wang, Tatsuo Sakai, Katsuhiko Asanuma, Yasuhiko Tomino

**Affiliations:** 1 Division of Nephrology, Department of Internal Medicine, Juntendo University School of Medicine, 2-1-1, Hongo, Bunkyo-ku, Tokyo, 113-8421 Japan; 2 Medical Innovation Center, Laboratory for Kidney Research (TMK Project), Kyoto University Graduate School of Medicine, Kyoto, Japan; 3 Department of Anatomy, Juntendo University School of Medicine, Tokyo, Japan; 4 Department of Nephrology, The First Hospital, China Medical University, Shenyang, China

**Keywords:** Podocyte, Podocin, Synaptopodin, IgA nephropathy, Puromycin aminonucleoside nephrosis rats

## Abstract

Podocytes serve as the final barrier to urinary protein loss through a highly specialized structure called a slit membrane and maintain foot process and glomerular basement membranes. Podocyte injury results in progressive glomerular damage and accelerates sclerotic changes, although the exact mechanism of podocyte injury is still obscure. We focus on the staining gap (podocin gap) defined as the staining difference between podocin and synaptopodin, which are normally located in the foot process. In puromycin aminonucleoside nephrosis rats, the podocin gap is significantly increased (*p* < 0.05) and podocin is translocated to the cytoplasm on days 7 and 14 but not on day 28. Surprisingly, the gap is also significantly increased (*p* < 0.05) in human kidney biopsy specimens of poor-prognosis IgA nephropathy patients. This suggests that the podocin gap could be a useful marker for classifying the prognosis of IgA nephropathy and indicating the translocation of podocin to the cytoplasm. Next, we find more evidence of podocin trafficking in podocytes where podocin merges with Rab5 in puromycin aminonucleoside nephrosis rats at day 14. In immunoelectron microscopy, the podocin positive area was significantly translocated from the foot process areas to the cytoplasm (*p*< 0.05) on days 7 and 14 in puromycin aminonucleoside nephrosis rats. Interestingly, podocin is also translocated to the cytoplasm in poor-prognosis human IgA nephropathy. In this paper, we demonstrate that the translocation of podocin by endocytosis could be a key traffic event of critical podocyte injury and that the podocin gap could indicate the prognosis of IgA nephropathy.

## Introduction

Podocytes are specialized epithelial cells constituting an essential part of the glomerular filtration barrier. Their interdigitated foot processes, connected by a slit diaphragm (SD), together with fenestrated endothelial cells and an intervening basement membrane, form the filtration barrier. The importance of podocyte integrity in the pathogenesis of nephrotic syndrome is best illustrated by the identification of human diseases causing mutations in genes encoding nephrin, podocin and CD2AP that span and stitch together foot processes of neighboring podocytes (Kestila et al. 1998; Ruotsalainen et al. 1999; Boute et al. 2000).

In the podocyte, podocin localizes in the SD, where it is assumed to act as an intercellular scaffold protein, assembling SD components in lipid raft-associated microdomains (Tryggvason et al. 2006; Huber et al. 2003). Podocin is a membrane-attached protein and it is predicted to form a hairpin-like structure, with both N- and C-terminuses residing in the cytoplasm (Schwarz et al. 2001). NPHS2 mutations cause several diseases with an interference of podocin intercellular trafficking (Roselli et al. 2004).

In mammalian cells, endocytosis is mediated via two principal routes, i.e., clathrin-mediated endocytosis (CME) and clathrin-independent, raft-mediated endocytosis (RME) (Le Roy and Wrana 2005). CME targets proteins to the early endosome, a sorting station directing vesicles to either recycling or degradation. Besides this classic CME pathway, RME has recently been the focus of intensive research, uncovering the new concept that the microdomain itself behaves as a vehicle for internalization. RME is generally defined by its clathrin independence, cholesterol sensitivity and a typical morphology of smooth invaginations (Mayor and Pagano 2007).

Shono et al. (2007) demonstrated that podocin co-localizes with the coxsaxie virus and adeno virus receptor (CAR) and with ZO-1 at the tight junction between foot processes in puromycin aminonucleoside (PA) nephrosis (PAN) rat kidneys and podocin facilitated the coalescence of lipid rafts containing CAR and makes dynamic cytoskeletal arrangement. They also demonstrated that podocin and CAR exhibit a diffuse punctate pattern throughout the cytoplasm in both proteins’ co-transfected COS-7 cells.

Regarding PAN, the intraperitoneal injection of PA to rats is an experimental model characterized by massive proteinuria and by marked morphological changes in podocytes, including the effacement of foot processes, their focal adhesion with Bowman’s capsules and the focal detachment from the GBM (Asanuma et al. 2003; Diamond et al. 1986; Nosaka et al. 1997). Thus, PA-induced nephrosis is regarded as an experimental model of human nephrotic syndrome and glomerulosclerosis.

In this paper, we demonstrate that podocin is translocated to the cytoplasm by endocytosis in both the PAN rat model and in poor-prognosis human immunoglobulin A nephropathy (IgAN) specimens using the difference in the staining of podocin and synaptopodin (synpo). This novel approach shows that podocin is translocated to the cytoplasm in the human nephropathy specimens and may help advance the understanding of podocin endocytosis.

## Materials and methods

### Antibodies

The monoclonal mouse anti-synaptopodin antibody (Progen, Heidelberg, Germany), Alexa 488 conjugated donkey anti-rabbit IgG antiserum (Invitrogen, CA, USA), Alexa 555 conjugated goat anti-mouse IgG antiserum (Invitrogen), mouse monoclonal Rab5 antibody (#50523 Abcam, Japan) and the 5-nm colloidal-gold-conjugated goat anti-rabbit IgG antiserum (heavy and light) (BB International, Cardiff, UK) were purchased for immunohistochemistry and/or immunoelectron microscopy. Polyclonal rabbit anti-podocin antiserum has been described previously (Schwarz et al. 2001).

### Experimental animals

Adult male Sprague–Dawley rats (weighing about 200 g) were obtained from the Sankyo laboratory service (Tokyo, Japan).

For PAN, a single dose (15 mg/100 g BW) of PA (Sigma, St. Louis, USA) was injected intraperitoneally into the rats to induce a nephrotic state, as described previously (Inokuchi et al. 1996; Kihara et al. 1995). These rats were housed under specific pathogen-free (SPF) conditions using individual metabolic cages with free access to standard chow and drinking water. Twenty-four-hour urine was collected once a week throughout the experiments. Urinary albumin, urinary total protein and creatinine were measured by the same methods as clinical examination. All experiments were performed according to the guidelines of the Committee on Animal Experiments of Juntendo University, Tokyo, Japan.

Three rats per group were sacrificed at 0, 4, 7, 14 and 28 days after PA injection. After the rats were anesthetized with Pentobarbital sodium (100 mg/kg; Dainippon Sumitomo Pharma, Osaka, Japan), they were perfused via the abdominal aorta with a PLP fixative buffer (4 % paraformaldehyde in 0.1 M lysine). After perfusional fixation, the kidneys were removed and processed for immunofluorescence and immunoelectron microscopy. Tissue slices were filled in Tissue-Tek O.C.T. compound (Sakura Finetek, USA), frozen in liquid nitrogen and then stored at −80 °C prior to immunofluorescence.

### Human tissue samples

For specimens of human IgA nephritis, tissue samples were obtained from the samples of diagnostic renal biopsies performed at Juntendo University Hospital with the permission of the Ethics Committee on Human Research of Juntendo University Faculty of Medicine. We investigated the samples from four groups of four patients, IgAN-good, IgAN-relatively-good (IgAN-r-good), IgAN-relatively-poor (IgAN-r-poor) and IgAN-poor, who had IgA nephropathy diagnosed according to the second guideline of IgA nephropathy (Tomino et al. 2003). As control human samples, we used biopsy samples from patients with minor glomerular abnormalities (*n* = 4).

### Immunofluorescence

For the immunofluorescent staining of rat kidneys, 4-μm-thick sections on sillan-coated slide glass were washed in PBS and incubated with a blocking solution. A double immunofluorescent staining for podocin and synpo was then performed and the secondary antibodies were incubated.

Human kidney biopsy specimens were stored at −80 °C for immunofluorescence. The 4-μm-thick sections were fixed with cold acetone for 5 min, washed with PBS, incubated with a blocking solution and then a double immunofluorescent staining for podocin and synpo was performed using the same methodology as with the PAN rats. These sections were photographed under a confocal laser microscope (Olympus FV1000, Tokyo, Japan).

To examine the podocin gap, or the staining gap between the area of podocin and synpo, at least 50 mid-sections of podocin and synpo areas were carefully measured using a digitizer KS-400 Imaging System as described previously (Tanimoto et al. 2004) and the podocin gap was calculated using the following formula:

$$ \mathrm{podocin}\;\mathrm{gap}\left(\%\right)=\left(\mathrm{podocin}\;\mathrm{fluorescent}\;\mathrm{staining}\;\mathrm{area}-\mathrm{synpo}\;\mathrm{fluorescent}\;\mathrm{staining}\;\mathrm{area}\right)/\mathrm{area}\;\mathrm{of}\;\mathrm{each}\;\mathrm{glomerulus}\times 100. $$

### Immunoelectron microscopy

Animals were perfused with physiological saline and subsequently with a PLP fixative. The perfused kidneys were cut into small pieces and immersed in the same fixative for 30 min. The samples were dehydrated with a graded series of ethanol and embedded in an LR-white resin (London Resin, Berkshire, UK) (Ichimura et al. 2009). Ultrathin gold sections of the LR-white-resin-embedded samples were produced with a diamond knife and transferred to nickel grids (150 mesh) that had been coated with a Formvar membrane. After blocking with a 1 % normal goat serum in PBS (pH 7.4), the sections were incubated overnight with the anti-podocin antiserum (1:50) diluted with 1 % BSA in PBS at 4 °C for 12 h. Subsequently, they were incubated with colloidal-gold-conjugated secondary antibodies (BB International) diluted 1:100 with 1 % BSA in PBS for 1 h at room temperature, contrasted with 4 % uranyl acetate for 5 min and observed with an H7100 transmission electron microsope (Hitachi High-Technologies, Tokyo, Japan). The primary antibodies were omitted from the incubation solution as a negative control and no non-specific staining of the secondary antibody was found in the kidney sections.

To confirm the alteration of localization of podocin in PAN rats, pictures from at least 15 podocyte cell bodies from each day were taken after an immunoelectron microscopic study. The immunogold podocin particles in the podocyte cell body were counted.

To examine the localization of podocin in human IgAN, tissues were taken with needle biopsy. Small cortical pieces were incubated in a PLP fixative buffer for a few hours and embedded in LR-white, the same as with the rat kidney tissues.

### Statistical analysis

All values are means ± SEM. Statistical significance (defined as *p* < 0.05) was evaluated using Stat View followed by the Fisher’s paired least significant difference *t* test.

## Results

### PAN rats showed significant proteinuria

**Fig. 1:**
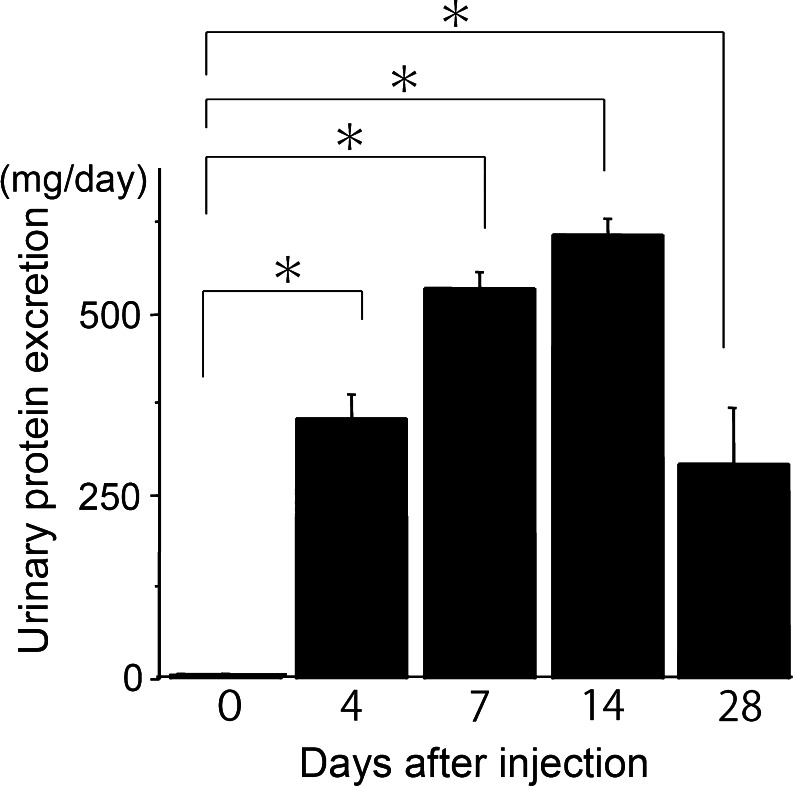
Puromycin aminonucleoside nephrosis rats showed significant proteinuria. Urinary protein was measured before and after the peritoneal injection of PA (15 mg/kg) to rats (*n* ≥ 4). On days 4, 7, 14 and 28 urinary protein was significantly increased (*p* < 0.001) (mean ± SE)

A single intraperitoneal injection of PAN produced overt proteinuria on days 4–28 (Fig. 1). We compared the levels of proteinuria at each time point from day 0. All time points, or days 4, 7, 14 and 28, showed significant proteinuria (*p* < 0.001). On day 28, the levels of proteinuria had decreased from the peak levels observed on day 14.

### PAN rats showed a difference in the staining of podocin and synpo on days 7 and 14

**Fig. 2:**
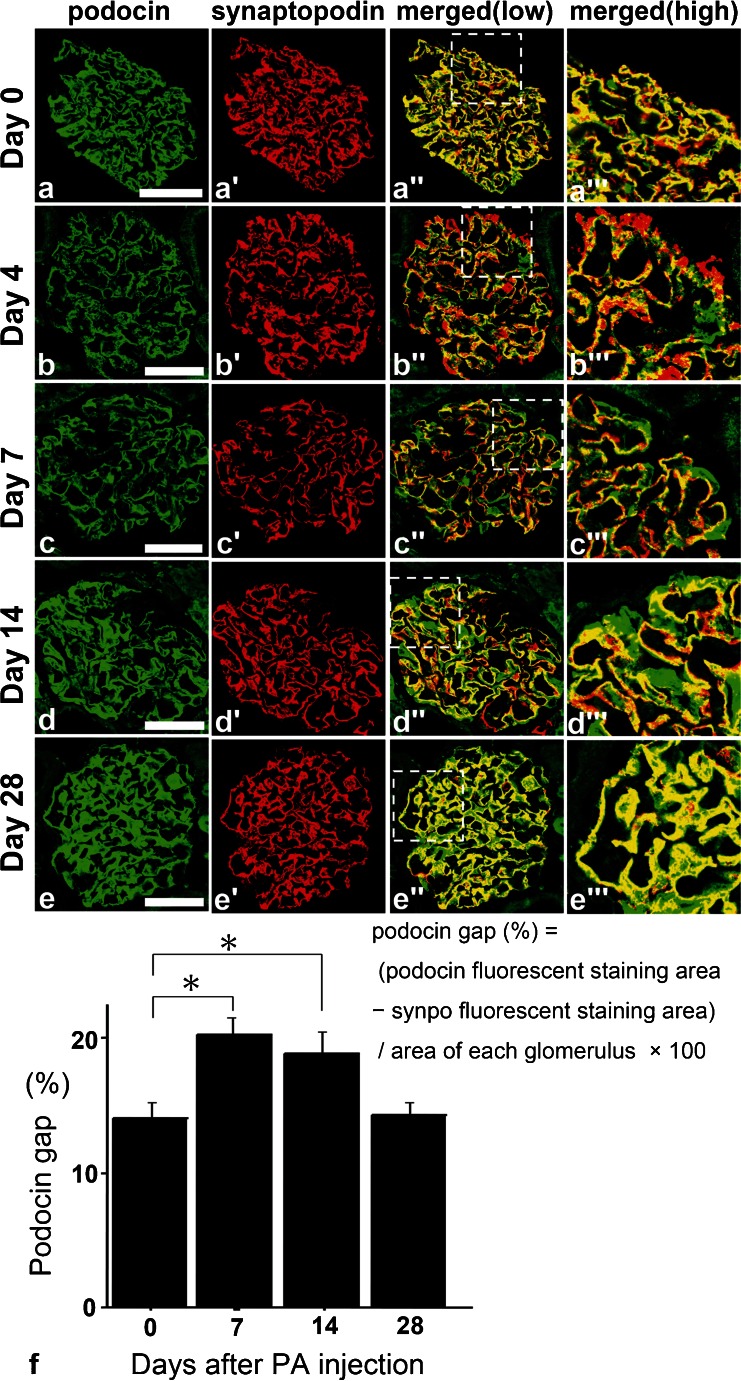
**a**–**e** Double fluorescence of podocin (*green*), synaptopodin (synpo) (*red*) and merged (*yellow*) in PAN rats. On day 4, the expression of podocin was decreased and the snypo area seemed larger than that of podocin but on days 7 and 14, the pattern of podocin staining had changed from a linear to cytoplasmic pattern. On day 28, the areas of both stainings were similar to control conditions (day 0). *Scale bars* (**a**–**e**) 50 μm. **f** Changes in the discrepancy staining area with podocin and synpo for each time course. We measured the podocin gap, the difference in the fluorescence staining area between podocin and synpo for each time point specimen for over 50 glomeruli using specific computer software (the mathematical formula is above this legend). KS400 software; see “Materials and methods”). On days 7 and 14, the gap was significantly increased (*n* ≥ 50) (*p* < 0.05) (mean ± SE)

Although on days 4 and 7 the expression of podocin and synpo were decreased, they were recovered on days 14 and 28 (Fig. 2a–e). The expression of podocin seemed to follow a linear pattern (similar to the glomerular basement membrane; GBM type) on days 4 and 7 and a podocyte cell body pattern on days 14 and 28. On the other hand, the linear staining of synpo did not change during the period from day 4 to day 28. On day 0, the staining patterns of podocin and synpo were almost matched (Fig. 2a). On days 7–14, the area of podocin seemed to be translocated to the cell body area from the foot process area; however, synpo stayed in the foot process area (Fig. 2c, d).

On day 28, their merged area was relocated to the foot process area (Fig. 2e). To confirm the difference of the podocin-synpo area, we calculated the podocin gap (see “Materials and methods”) with the use of computer software by subtracting the synpo area from the podocin area and dividing the total by the glomerulus area (Fig. 2f). On days 7–14, the podocin gap was significantly increased (*p* < 0.05) and on day 28, the area returned to the same size as measured on day 0.

### Podocin was translocated to the cytoplasm of PAN rats at day 14

**Fig. 3:**
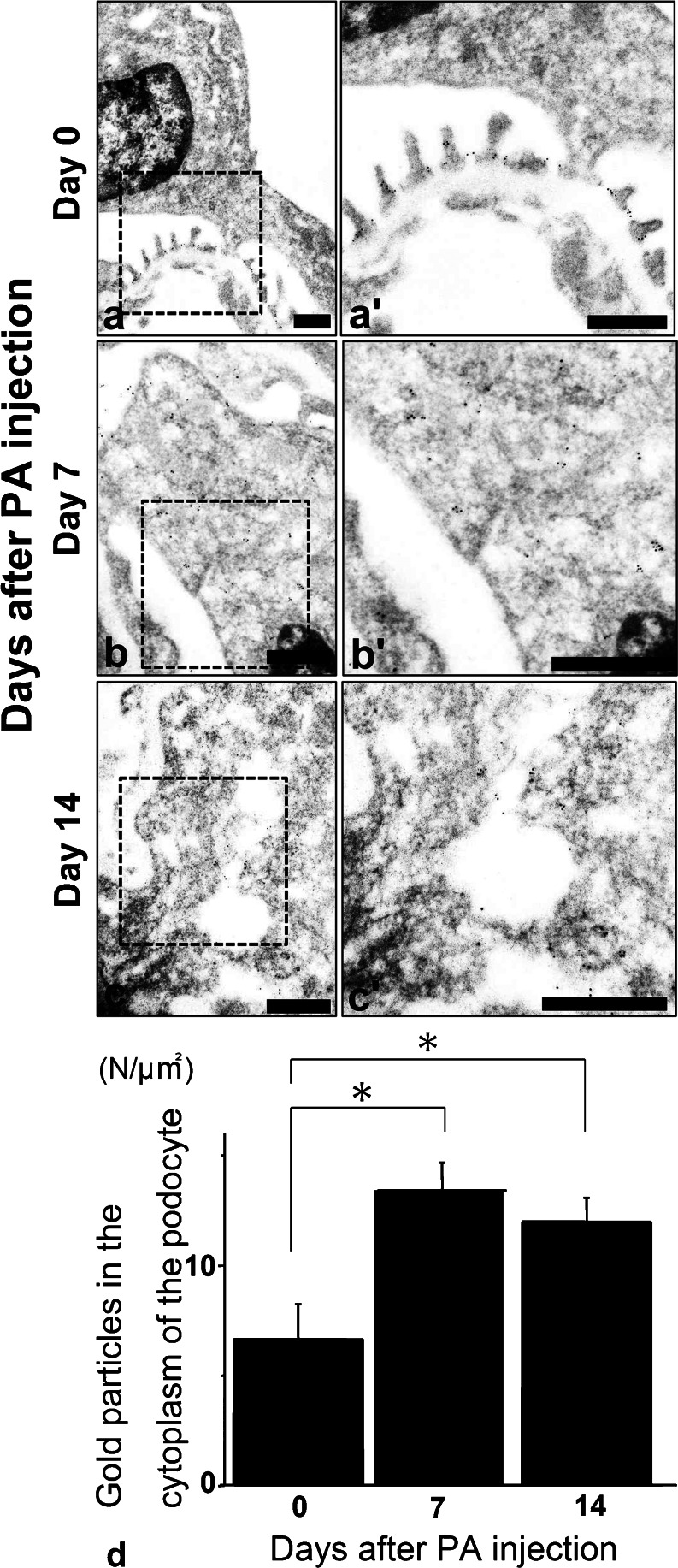
**a**–**c** Immunoelectron microscopy of podocin in PAN rats. Podocin moved from the slit diaphragm area to the cytoplasmic area as a form of vesicles at day 14 in the podocytes. *Scale bars* (**a**–**c**, **a’**–**c’**)50 nm. *Left* low-magnification images (×8000–×20,000) and *right* high-magnification images (×10,000–×50,000). **d** Podocin significantly moved to the cytoplasm of podocytes on days 7 and 14 in PAN rats. The number of gold particles (podocin) was calculated in the cytoplasmic area of podocytes. On days 7 and 14, podocin was significantly increased in the cytoplasmic area in podocytes (*n* ≥ 15) (*p* < 0.05) (mean ± SE)

In the immunoelectron microscopy of PAN rats, immunoreactive podocin was recognized by gold particles (Fig. 3a, b, c: low magnification ×8000–×20,000, 3a’, b’, c’: high magnification ×10,000–×50,000). On day 0, podocin was recognized at the slit diaphragm insertion sites of foot processes (Fig. 3a, day 0). On day 7, the structure of the slit diaphragm was destroyed and the podocin was translocated to the cell bodies of the podocytes (Fig. 3b, day 7). On day 14, through staining, podocin was seen in the vesicular structures in the cell bodies of podocytes (Fig. 3c, day 14). These data indicate that the podocin was translocated to the cell body, likely by endocytosis.

By counting the number of gold particles in the cytoplasm of the podocytes, we could detect significantly more podocin in the cytoplasm on days 7 and 14, as compared to day 0 (Fig. 3d, *p* < 0.05).

### The podocin gap was significantly increased in IgAN-poor prognosis specimens

**Fig. 4:**
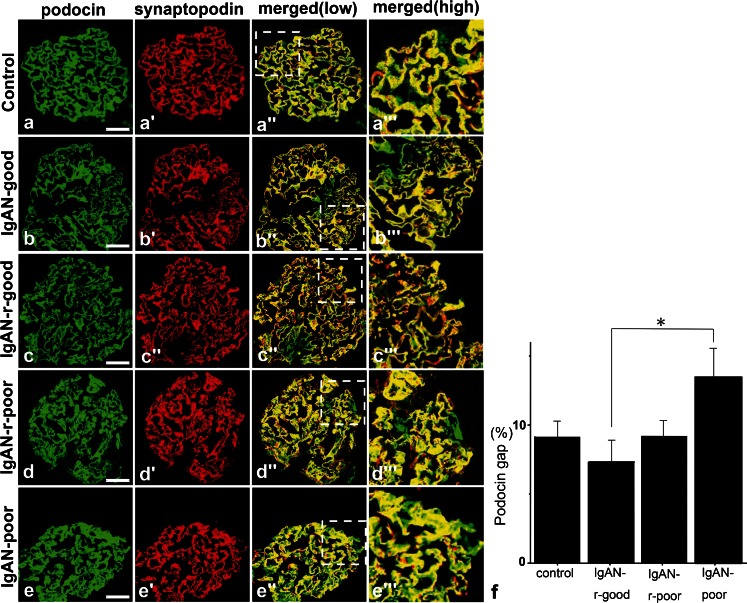
**a**–**e** Double fluorescence of podocin (*green*), synpo (*red*) and merged (*yellow*) in IgA Nephropathy. In IgAN-good, −r-good and -r-poor specimens, the staining areas of podocin and synpo were almost the same but the IgAN-poor podocin area (not merged with synpo, *green*) was larger than that of the other groups and the staining pattern had changed from a linear type to cell body type. *Scale bars* (**a**–**e**) 50 μm. **f** The changes in the discrepancy staining area with podocin and synpo in each prognosis categories of IgAN. We measured the podocin gap (see Fig. 2f). In IgAN-r-good specimens, the gap was decreased as compared with that of the control but interestingly, in the IgAN-poor group, the gap was significantly increased (*p* < 0.05) compared with that of IgAN-r-good control specimens, i.e., minor glomerular abnormality (*n* ≥ 4) (mean ± SE)

Each of the four IgAN groups consisted in four samples that were stained with podocin and synpo (Fig. 4a–e). In the IgAN-good group, the podocin and synpo stained areas were consistently similar to day 0 in PAN rats (Fig. 4b, IgAN-good). In the IgAN-r-good group, the level of expression was decreased but both of them were merged completely (Fig. 4c, IgAN-r-good). In the IgAN-r-poor group, there was no evident change from the IgAN-good group (Fig. 4d, IgAN-r-poor). Surprisingly, in the IgAN-poor group, podocin was stained in the cell bodies of podocytes and there was a clear difference in the staining pattern between podocin and synpo. This result suggests the translocation of podocin to the cytoplasm. We also measured the podocin gap in each human biopsy sample from IgAN (Fig. 4f) using the previously mentioned software. The podocin gap was not altered in the IgAN-r-good and IgAN-r-poor groups when compared to the control group. However, in the IgAN-poor group it was significantly increased (*p* < 0.05) when compared to the area of the IgAN-r-good group, indicating that podocin translocated to the cell bodies of podocytes.

### Podocin was translocated to the cytoplasm in IgAN poor prognosis specimens

**Fig. 5:**
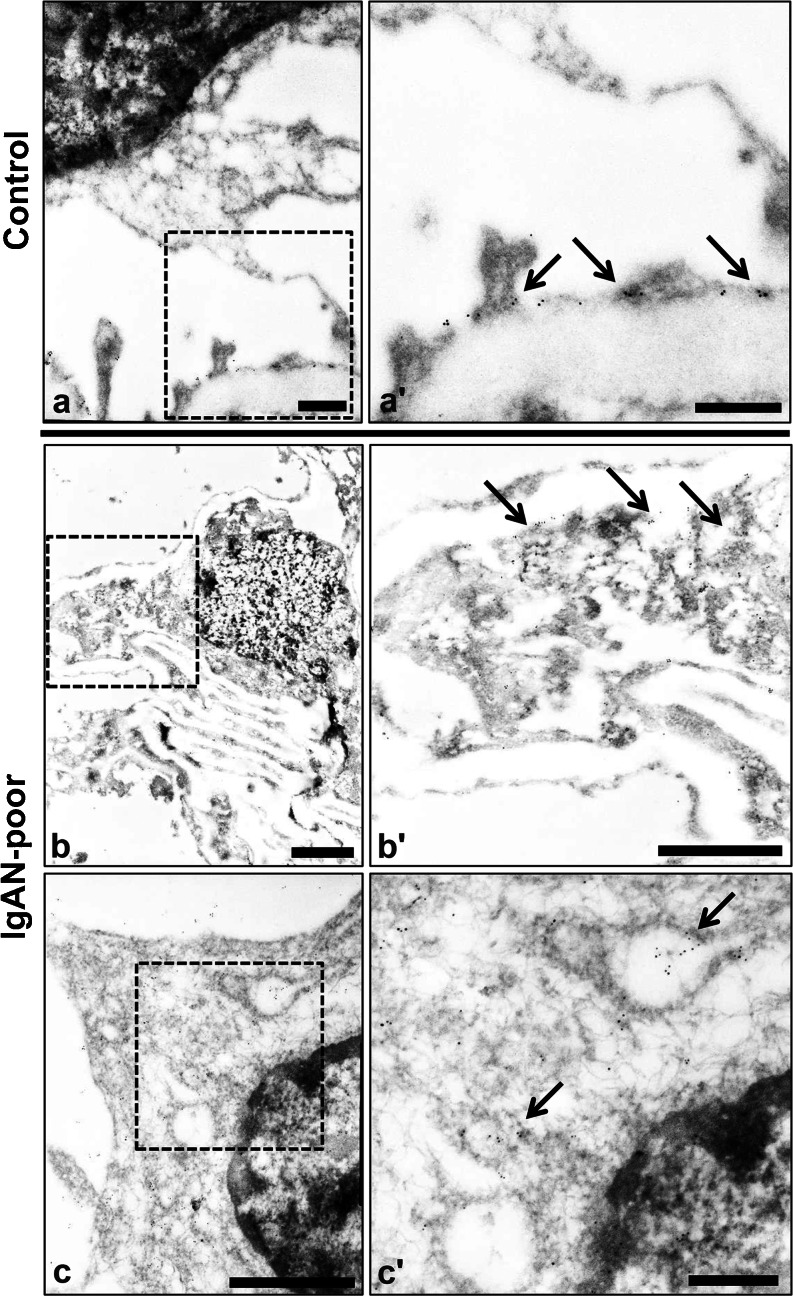
**a**–**c** Immunoelectron microscopy of podocin in the control and IgAN-poor group. Podocin was translocated from the slit diaphragm area to the cytoplasmic area in the IgAN-poor group. *Scale bars* (**a**, **a’**, **c’**) 500 nm, (**b**, **b’**, **c**) 2 μm. Normal glomerulus: minor glomerular abnormalities. *Left* low-magnification images (×8000), *right* high- magnification images (×30,000)

We next performed immunoelectron microscopy using an anti-podocin antibody in kidney biopsy specimens of minor glomerular injury and IgAN-poor specimens (Fig. 5a–c). Podocin was located in the SD area of the control specimen. On the other hand, in the IgAN-poor biopsy specimens, the structure of the foot processes was destroyed and podocin was translocated to the cytoplasm area (Fig. 5b, c, IgAN-poor).

### Podocin was translocated to the cytoplasm by the endocytosis pathway

**Fig. 6:**
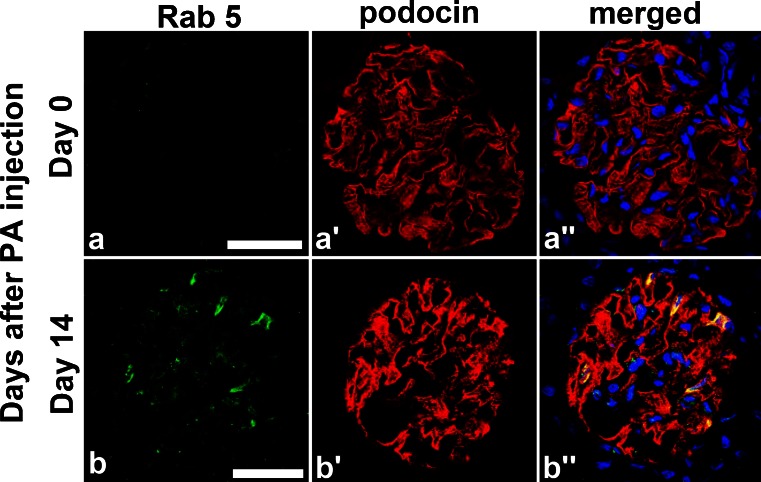
**a**, **b** Podocin and Rab5 merged in samples from day 14 PAN rats. We performed double staining of podocin and Rab, which is a specific early endosome marker, in day 14 PAN rats specimens and detected several locations where podocin clearly merged with Rab5 (*arrow*). This suggests podocin translocation to the cytoplasm by endocytosis. *Scale bars* 50 μm

As mentioned above, at day 14 of PAN rats, podocin was translocated to the cell body area from foot processes. To check this translocation mechanism, we stained PAN rats specimens at day 14 with podocin and Rab5 (early endosome marker) (Fig. 6a, b). Several podocytes were merged with Rab5 and this result indicated that podocin was translocated to cytoplasm by the endocytosis pathway.

### Podocin was not translocated to the cytoplasm in minimal change disease

**Fig. 7:**
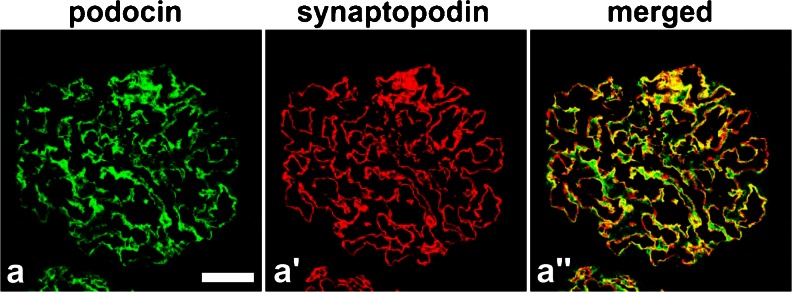
In human MCD, podocin and synpo clearly merged and there was no significant podocin gap. Evaluation of the podocin gap in human MCD specimens showed that podocin and synpo stained the same foot process area. We did not find any evidence of podocin translocation. *Scale bar* (**a**) 50mm

We evaluated the podocin gap in human minimal change disease (MCD) biopsy specimens. Podocin and synpo merged clearly and no podocin gap could be detected in MCD (Fig. 7).

## Discussion

In this paper, we demonstrated that podocin was translocated from the SD area to the cytoplasm area by the endocytosis pathway in PAN rats and in the IgAN-poor prognosis group. We defined the podocin gap as follows: the difference in the staining pattern between podocin and synpo, which are foot processes proteins. Podocin is especially prevalent in the SD but synpo is located beside actin bundles in the foot processes (Shirato et al. 1996). As we demonstrated before, synpo is a critical protein for podocytes and for organizing their structure (Asanuma et al. 2006). In the absence of synpo, the podocytes lose essential stress fibers and their cell bodies shrink so as to resemble a fibroblast. On the other hand, SD proteins were naturally translocated from SD to the cytoplasm and kept their structure (Soda and Ishibe 2013). Within a diseased condition, the endocytosis system of SD proteins may become damaged and the damage may accelerate podocyte injury. Thus, we hypothesize that the difference of localization between podocin and synpo may occur during podocyte injury. During proteinuria conditions, the difference between the localization of podocin and synpo was significantly more evident than during normal conditions. To focus on the podocin gap, we checked the localization of podocin and therefore we also demonstrated that podocin was translocated to the cytoplasmic area with immune electron microscopy. Although podocin was translocated to the cytoplasm, it does not correlate to the level of proteinuria. Actually, the podocin gap was not found in either MCD, IgAN-r-good or IgAN-r-poor specimens. We estimated that the translocation of podocin happened in severe podocyte injury.

Soda et al. (2012) reported that double knocked-out dynamin-1 and -2 mice, synaptojanin knocked-out mice and triple endophilin-1, -2, and -3 knocked-out mice showed significant proteinuria and foot process effacement. They further demonstrated that synaptojanin and endophilin, which are functional partners of dynamin in synaptic vesicle endocytosis at neural synapses, were critically implicated in the development of the permeability barrier of kidneys. This paper indicated that the system of endocytosis in the podocytes is a critical phenomenon and that it is tightly connected to proteinuria and foot process effacement (FPE), further worsening the podocyte injury (these knocked-out mice displayed a lethal kidney failure).

Recent evidence suggests that endocytosis may play a vital role in the internalization and recycling of nephrin (Tossidou et al. 2010). Phosphorylation (Quack et al. 2006) and the stimulation of high glucose (Tossidou et al. 2010) may initiate nephrin endocytosis and depend on CIN/RukL, the homologue of CD2AP (Tossidou et al. 2012). Focusing on endocytosis, Qin et al. (2009) demonstrated that a raft-mediated endocytic pathway internalizes nephrin and Godel et al. (2013) demonstrated that the trafficking of podocin is dependent on the raft-mediated, non-conventional endocytic pathway. This seems to be consistent with the suggestion for endocytic trafficking of other prohibitin-domain proteins (Langhorst et al. 2005). Intriguingly, the podocin-related protein flotillin-1 defines a clathrin-independent endocytic pathway (Glebov et al. 2006), which could lead to the hypothesis that podocin not only assembles members of the slit diaphragm but also orchestrates its internalization via a self-defined pathway (Godel et al. 2013). In this study, we demonstrated that podocin merged with Rab5 in PAN rats at day 14. At the same time, the podocin gap was significantly elevated, indicating that podocin was translocated to the cytoplasm by the endocytosis pathway. Because Rab5 is a typical CME marker, it shows evidence that podocin interacts with the CME pathway.

The Wiggins group demonstrated that a single dose of PA caused podocytes depletion that results in minor glomerular sclerosis (Kim et al. 2002). As a podocyte injury model, we used PAN rats with a single peritoneal PA injection with the same methods of the Wiggins’ group. For our model of podocyte injury, we used PAN rats prepared with a single peritoneal PA injection, just as the Wiggins’ group used for their experiment. PA induces oxidant injury in cells via the xanthine oxidase pathway and it was used as a similar model of MCD and focal segmental nephron sclerosis in human (Seckin et al. 2012). We evaluated PAN rats samples, which showed podocytes detachment and glomerular sclerosis. Clinically, MCD does not show such podocyte change without FPE, so we used this model as a podocytes injury model. We also tried to stain the podocin gap in human MCD; however, there was no difference in the podocin and synpo staining (Fig. 7).

Recently, two papers demonstrated that FPE has been interpreted as a protective response of podocytes in danger of detachment (Kriz et al. 2013; Kriz and Lemley 2014). This FPE is typically observed in MCD and in other glomerulonephritis. Similarly, PAN rats and IgAN patients also show these pathological findings. The point is that the podocin gap was observed only on PAN days 7, 14 and IgAN-poor. These pathological findings show severe glomerular damage and podocytes injury. Compared to FPE, the podocin gap was only seen in severe conditions and we thought it could be a predictive factor to assess the IgAN.

IgAN is the most common primary glomerulonephritis. An often insidious progression to end-stage kidney disease in 25–40 % of cases is accompanied by the development of glomerulosclerosis (Rychlik et al. 1999). It is characterized by the mesangial deposition of IgA, associated with mesangial cell proliferation and mesangial matrix expansion. In addition to these common histologic abnormalities, other glomerular abnormalities, such as segmental sclerosis, crescent formation and adhesion to the bowman capsules, are detected. Other indicators, such as the number of podocytes per glomerulus, might serve as a parameter of podocyte injury and provide prognostic information for patients with IgAN (Hishiki et al. 2001). Lemley et al. (2002) reported that podocytopenia is associated with increasing disease severity in IgAN. The clinical course of IgAN is variable. The prevalence of clinically silent IgAN may be surprising high; in a Japanese study, 16 % of donor kidneys had glomerular IgA deposits and nearly 2 % exhibited mesangioproliferative changes with C3 deposits characteristic of IgAN (Suzuki et al. 2003). Mesangial IgA is exclusively of the IgA1 subclass and is deficient in galactose (Suzuki et al. 2011). Besides these findings, the proceeding mechanisms of IgAN are still obscure. Therefore, we focused on the difference in pathological characterizations of IgAN. On this basis, we divided IgAN samples into 4 categories, following the indicators of the Japan Committee of IgAN 2002 and assessed each specimen. Only the poor prognosis specimens showed a significant difference in the podocin gap and this showed a translocation of podocin to cytoplasm by endocytosis. Based on these results, we hypothesize that podocin traffic may lead to severe podocyte injury or may happen in severely injured podocytes. With these results, we postulate that podocin traffic may also predict the prognosis of an IgAN disease course.

When evaluating IgAN, the patient’s samples are very important for estimating their prognosis; however, at present, we do not have a specific staining reference. If endocytosis is more fully understood, the movement of some proteins may be the key to knowing the turning point between reversible change and irreversible change.

In the future, the role of podocin traffic may help shed light on the mechanism of podocyte injury and indicate a medical approach to prevent the advancement of the disease course.
